# Effects, Safety, and Treatment Experience of Advanced Hybrid Closed-Loop Systems in Clinical Practice Among Adults Living With Type 1 Diabetes

**DOI:** 10.1177/19322968241242386

**Published:** 2024-04-17

**Authors:** Ramanjit Singh, Henrik Imberg, Shilan Seyed Ahmadi, Sara Hallström, Johan Jendle, Bengt-Olov Tengmark, Anna Folino, Ekström Marie, Marcus Lind

**Affiliations:** 1 Department of Medicine, Sahlgrenska University Hospital, Gothenburg, Sweden; 2 Department of Molecular and Clinical Medicine, Sahlgrenska Academy, University of Gothenburg, Gothenburg, Sweden; 3 Department of Mathematical Sciences, Chalmers University of Technology and University of Gothenburg, Gothenburg, Sweden; 4 Statistiska Konsultgruppen, Gothenburg, Sweden; 5 Faculty of Medicine and Health, School of Medical Science, Örebro University, Örebro, Sweden; 6 Citydiabetes, Stockholm, Sweden; 7 Department of Medicine and Emergency, Sahlgrenska University Hospital/Mölndal Hospital, Gothenburg, Sweden; 8 Department of Medicine, NU Hospital Group, Uddevalla, Sweden

**Keywords:** type 1 diabetes, time in range, advanced hybrid closed loop, patient-reported outcome

## Abstract

**Background:**

There are few studies providing a more comprehensive picture of advanced hybrid closed-loop (AHCL) systems in clinical practice. The aim was to evaluate the effects of the AHCL systems, Tandem^®^ t: slim X2™ with Control IQ™, and MiniMed™ 780G, on glucose control, safety, treatment satisfaction, and practical barriers for individuals with type 1 diabetes.

**Method:**

One hundred forty-two randomly selected adults with type 1 diabetes at six diabetes outpatient clinics in Sweden at any time treated with either the Tandem Control IQ (TCIQ) or the MiniMed 780G system were included. Glycated hemoglobin A1c (HbA1c) and glucose metrics were evaluated. Treatment satisfaction and practical barriers were examined via questionnaires.

**Results:**

Mean age was 42 years, median follow-up was 1.7 years, 58 (40.8%) were females, 65% used the TCIQ system. Glycated hemoglobin A1c was reduced by 0.6% (6.8 mmol/mol; 95% confidence interval [CI] = 0.5-0.8% [5.3-8.2 mmol/mol]; *P* < .001), from 7.3% to 6.7% (57-50 mmol/mol). Time in range (TIR) increased with 14.5% from 57.0% to 71.5% (95% CI = 12.2%-16.9%; *P* < .001). Time below range (TBR) (<70 mg/dL, <3.9 mmol/L) decreased from 3.8% to 1.6% (*P* < .001). The standard deviation of glucose values was reduced from 61 to 51 mg/dL (3.4-2.9 mmol/L, *P* < .001) and the coefficient of variation from 35% to 33% (*P* < .001). Treatment satisfaction increased, score 14.8 on the Diabetes Treatment Satisfaction Questionnaire (DTSQ) (change version ranging from −18 to 18, *P* < .001). Four severe hypoglycemia events were detected and no cases of ketoacidosis. Skin problems were experienced by 32.4% of the study population.

**Conclusions:**

Advanced hybrid closed-loop systems improve glucose control with a reasonable safety profile and high treatment satisfaction. Skin problems are common adverse events.

## Introduction

It is well established that higher blood glucose levels increase the risk of future microvascular complications and cardiovascular disease.^1-8^ Advanced hybrid closed-loop (AHCL) systems employ specific algorithms allowing continuous automated basal insulin delivery and correction boluses in response to varying interstitial glucose levels measured by continuous glucose monitoring (CGM) and thus aim to minimize fluctuations outside of the targeted glucose range. In 2016, the first AHCL system, MiniMed™ 670G, was introduced and subsequently upgraded to the MiniMed™ 780G (M780) system a few years later.^9^ A second AHCL system, Tandem^®^ t:slim X2™ with Control IQ™ (TCIQ), was approved by the US Food and Drug Administration (FDA) in 2019 after a six-month randomized, multicenter trial demonstrated improved mean time in range (TIR), from 61% to 71%, compared with sensor-augmented pumps (SAPs) without automated insulin delivery.^10^

Currently, few reports^11,12^ have studied outcomes of AHCL systems in a real-world setting evaluating effects on glucose metrics, treatment satisfaction, as well as practical barriers and safety. In one large prospective study,^11^ comparison of minimum 30-day CGM data pre-activation and post-activation of the TCIQ system demonstrated a 10% improvement in median percentage TIR. Similar improvements in TIR with the same AHCL system were demonstrated in children and young adults at 6 months after system use commenced thus doubling the number of individuals reaching TIR goals (> 70%).^13^

To date, several real-world evidence reports^13-17^ have focused on effects on glycemic control without including patient-reported outcomes (PROs). Studies have in many instances not evaluated safety since CGM data have been downloaded from ongoing pump users, with safety data of ketoacidosis, severe hypoglycemia’s, and injuries related to AHCL system therapy and experiences of practical barriers generally lacking.

In Sweden, generally the AHCL systems TCIQ and M780 are those used in clinical practice. In this multicenter retrospective study, we aimed to evaluate real-world outcomes in adults living with type 1 diabetes treated with the TCIQ or the M780 system regarding metabolic control, safety profile, practical barriers, as well as treatment satisfaction.

## Methods

### Study Design and Data Collection

Individuals at any time treated with the Tandem Control IQ (TCIQ) or the MiniMed 780G (M780) system were identified through locally used quality registers. Patients were thereafter randomly selected for participation and informed about the study via telephone or at clinical visits. Besides previous or current treatment with the TCIQ or M780 system, inclusion criteria consisted of age ≥ 18 years and diagnosis of type 1 diabetes. Exclusion criteria were diagnosis of type 2 diabetes, diabetes duration less than one-year, prior treatment with the MiniMed 670G system, or pregnancy six months prior to or during treatment with the TCIQ or M780 system.

Patients were recruited at six diabetes outpatient clinics in Sweden, which included Sahlgrenska University Hospital/Östra and Sahlgrenska University Hospital/Mölndal in Gothenburg, NU-Hospital Group in Trollhättan and Uddevalla, Lidköping Hospital, Kungälv Hospital, and CityDiabetes Serafen in Stockholm. The study was approved by the Swedish Ethical Review Authority (diary number 2023-00651-02). Written informed consent was obtained from all patients.

### Study Procedures

Annual glycated hemoglobin A1c (HbA1c) values (±6 months) 5 years prior to beginning use of the TCIQ or the M780 system as well as quarterly 1 year prior to beginning use were collected. In addition, the last available HbA1c value before starting treatment was recorded. After starting treatment, HbA1c values at one month and quarterly thereafter were documented. These values were gathered at identical intervals during both Basal-IQ and Control IQ, respectively, for the Tandem system.

Continuous glucose monitoring data were collected by similar procedures and at similar time intervals as HbA1c. Four weeks of CGM data were collected at each time point except for one month after starting treatment where two weeks of CGM data were considered sufficient. If desirable duration of CGM-data was not available, the longest period of available data was chosen (corresponding to at least five days of data with a minimum of 70% CGM activity time).

The Diabetes Treatment Satisfaction Questionnaire change version (DTSQc)^18^ and Diabetes Treatment Satisfaction Questionnaire status version (DTSQs)^19^ were used for assessment of satisfaction with the current treatment in comparison to previous treatment. The DTSQc scores range from −18 to +18, with higher scores implying greater satisfaction with current treatment in comparison with earlier treatment. A panel of diabetes nurses and physicians experienced in treating type 1 diabetes constructed a questionnaire consisting of 23 questions focusing on treatment experience, including potential management difficulties, such as sensor problems and adverse events, including any long-term disabilities and hospitalizations related to the AHCL systems. Scores in 15 of these questions range from 1 to 10 with higher scores indicating greater agreement with the statement given in the question. Other inquired adverse events included severe hypoglycemia (defined as hypoglycemia requiring assistance and/or leading to unconsciousness) and ketoacidosis (defined as a pH value < 7.3 in combination with other clinical findings that support the diagnosis, specifically typical symptoms, plasma glucose level > 14.0 mmol/L (> 250 mg/dL), and blood ketones > 3.0 mmol/L (> 54 mg/dL). Questionnaires were filled out by participants after giving informed consent.

The primary endpoint was the change in percentage of TIR (70-180 mg/dL, 3.9-10.0 mmol/L) between the last available measurement before starting treatment and the last available measurement after initiation of TCIQ or the M780 system. Secondary endpoints included change in time below range (TBR) (< 54 mg/dL, < 3.0 mmol/L), time above range (TAR) (> 250 mg/dL, > 13.9 mmol/L), change in HbA1c, change in percentage of patients obtaining HbA1c ≤ 7.0% (≤ 53.0 mmol/mol), change in glycemic variability measured by the standard deviation (SD) of glucose values, and DTSQc total score. Exploratory endpoints included change in TBR (< 70 mg/dL, < 3.9 mmol/L), change in TAR (> 180 mg/dL, > 10.0 mmol/L), change in glycemic variability measured by the coefficient of variation (CV) of glucose values, change in time in target (TIT) (70-144 mg/dL, 3.9-8.0 mmol/L), change in mean glucose level, change in glucose management indicator (GMI), change in percentage of patients with TIR ≥ 70%, and change in percentage of patients with HbA1c greater than or equal to 8% (64.0 mmol/mol) and 9% (75.0 mmol/mol), respectively. In a post hoc analysis, we also evaluated the glycemia risk index, including a hypoglycemia component, hyperglycemia component, and total index ranging from 0 to 100.^20^ In accordance with the primary endpoints, the last available measurement before starting treatment was compared with the last available measurement after initiation of TCIQ or the M780 system regarding both secondary and exploratory endpoints.

### Statistical Analysis

Descriptive statistics are presented as mean and SD for numeric variables and numbers and percentages for categorical variables.

Changes from baseline to follow-up were analyzed using repeated measures regression modeling to account for missing data. An unstructured covariance matrix between pairs of measurements from the same individual was used. This is equivalent to the paired *t* test in cases where complete data are available. Sensitivity analyses using paired *t* test on complete cases were also performed. The Diabetes Treatment Satisfaction Questionnaire change version was analyzed using the one-sample *t* test. Patient-reported outcomes and adverse events are presented descriptively.

A sample size of *n* = 140 individuals was needed to demonstrate an improvement of 3% (approximately 45 minutes per day) in TIR from baseline to follow-up, assuming a SD of 14%, correlation = 0.60 between TIR at baseline to follow-up, 80% power, two-sided test, significance level α = 5%.

Comparisons between the TCIQ and M780 systems with respect to primary, secondary, and exploratory endpoints were performed using analysis of covariance (ANCOVA), adjusting for baseline values. The mean differences in DTSQ total score, status and change versions, were analyzed using Welch’s *t* test. Adjustments for age, sex, diabetes duration, and previous treatment with multiple daily injections (MDIs) or insulin pump were performed using ANCOVA. Other PROs were compared between groups using Fisher’s exact test for binary variables and the Mann-Whitney *U* test for ordinal and numeric variables. These comparisons were adjusted for the aforementioned potential confounders using Spearman’s partial correlation analyses, which correspond to covariate-adjustment in linear regression on rank-transformed data.

All tests were two-sided and conducted at the 5% significance level. Statistical analyses were performed using SAS/STAT^®^ Software, Version 9.4 of the SAS System for Windows (SAS Institute Inc., Cary, NC).

### Data Availability Statement

The data sets generated during and/or analyzed in the current study are available from the corresponding author upon reasonable request.

## Results

### Baseline Characteristics

**Figure 1.**
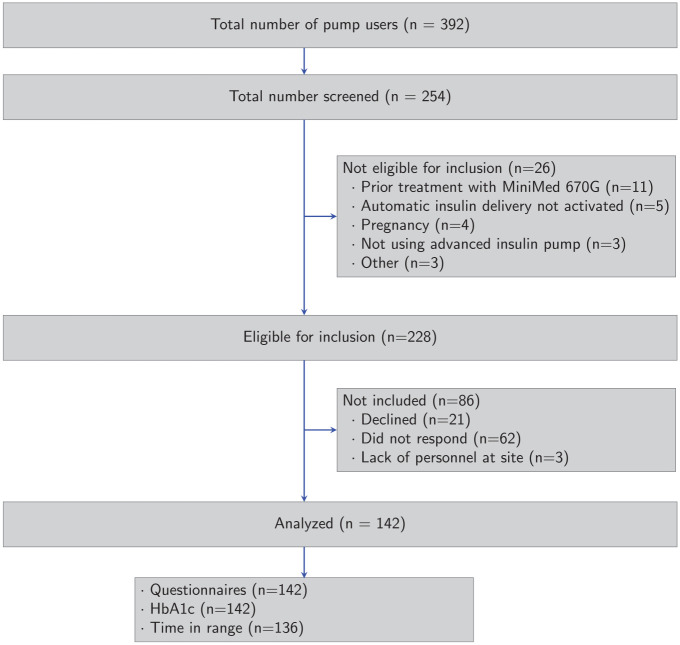
Flow chart of the study cohort according to CONSORT guidelines, including number of subjects screened, included, and analyzed. Number of subjects analyzed is the number of individuals with questionnaire data, HbA1c, and CGM-data, respectively, at baseline (prior to start of AHCL therapy) or follow-up.

A total of 392 AHCL pump users were identified until February 2023, of which 254 were screened for participation. Among these, 26 were found illegible for inclusion due to prior treatment with MiniMed 670G (*n* = 11), automatic insulin delivery not activated (*n* = 5), pregnancy (*n* = 4), not using AHCL (*n* = 3), or other reasons (*n* = 3) (Figure 1). In the end, 142 patients were included in the study, of which 92 (65%) were users of the TCIQ and 50 (35%) of the MiniMed 780G (M780) system. Mean age for patients using the TCIQ was 40 years (SD = 14) and 45 years (SD = 14) for the M780 system (Table 1). Among patients using the M780 system, prior use of the MiniMed 640G system was more common in comparison with users of the TCIQ (80% and 13%, respectively). Mean HbA1c prior to start of treatment was 7.4% (57.2 mmol/mol) (SD = 1.2%; 12.7 mmol/mol) in the TCIQ group and 7.2% (55.7 mmol/mol) (SD = 0.9%; 9.8 mmol/mol) in the M780 group. Mean TIR was 53% (SD = 18%) and 63% (SD = 15%) in the TCIQ and M780 groups, respectively. Median (interquartile range [IQR]) follow-up time was 1.7 (1.1-2.6) years.

**Table 1.** Baseline Characteristics of the Study Cohort Presented Separately for Persons Living With Type I Diabetes Initiating Treatment With the MiniMed 780G and Tandem Control IQ System.

Variable	Total (*n* = 142)	Tandem control IQ (*n* = 92)	MiniMed 780G (*n* = 50)
Age (years)	41.9 (14.2)	40.4 (14.0)	44.7 (14.1)
Female sex	58 (40.8)	41 (44.6)	17 (34.0)
Diabetes duration (years)	23.3 (12.3)	21.5 (11.8)	26.6 (12.7)
HbA1c (%)	7.3 (1.1)	7.4 (1.2)	7.2 (0.9)
HbA1c (mmol/mol)	56.6 (11.7)	57.2 (12.7)	55.7 (9.8)
Percentage of time in range, 70-180 mg/dL	57.0 (17.4)	53.1 (17.6)	62.6 (15.2)
Smoking			
*Current*	6 (4.5)	2 (2.3)	4 (8.3)
*Former*	24 (17.9)	17 (19.8)	7 (14.6)
*Never*	104 (77.6)	67 (77.9)	37 (77.1)
BMI (kg/m^2^)	27.5 (4.5)	27.3 (4.8)	27.8 (4.0)
Creatinine (µmol/L)	78.4 (27.6)	73.6 (20.0)	86.5 (36.0)
Systolic blood pressure (mmHg)	125.5 (12.8)	124.5 (13.0)	127.3 (12.5)
Diastolic blood pressure (mmHg)	73.6 (8.8)	74.1 (9.1)	72.9 (8.5)
Lipid-lowering treatment	67 (75.3)	37 (69.8)	30 (83.3)
Antihypertensive treatment	39 (43.8)	20 (37.7)	19 (52.8)
Lipid-lowering and antihypertensive treatment	31 (34.8)	14 (26.4)	17 (47.2)
Macroalbuminuria	4 (3.0)	1 (1.2)	3 (6.4)
Microalbuminuria	5 (3.8)	4 (4.7)	1 (2.1)
Retinopathy			
*Severe*	22 (16.3)	9 (10.5)	13 (26.5)
*Moderate*	19 (14.1)	11 (12.8)	8 (16.3)
*Mild*	49 (36.3)	32 (37.2)	17 (34.7)
Earlier MI, CABG, or PCI	4 (3.0)	1 (1.2)	3 (6.3)
Previous amputation	2 (1.5)	1 (1.2)	1 (2.1)
Treatment prior to closed-loop pump			
*MDI*	38 (26.8)	31 (33.7)	7 (14.0)
*Insulin pump*	104 (73.2)	61 (66.3)	43 (86.0)
*MiniMed 640G*	52 (36.6)	12 (13.0)	40 (80.0)

Categorical variables are reported as number (percentage).

Continuous variables are reported as mean (standard deviation).

Abbreviations: BMI, body mass index; CABG, coronary artery bypass graft; HbA1c, glycated hemoglobin A1c; MDI, multiple daily injection; MI, myocardial infarction; PCI, percutaneous coronary intervention.

### Effects on Glucose Control

**Figure 2.**
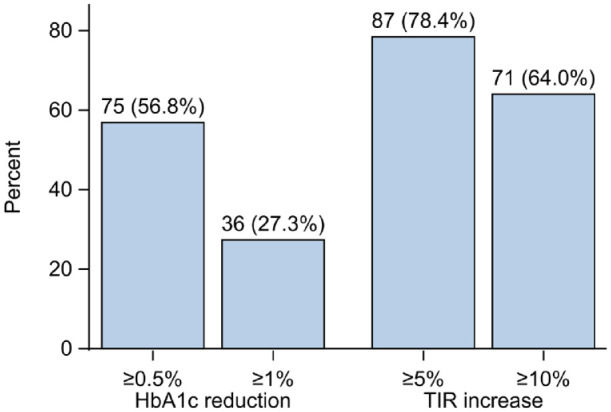
Proportion of persons with an improvement in HbA1c of 0.5% (5.5 mmol/mol) and 1% (11 mmol/mol) and time in range of 5% and 10%. Numbers and percentages are presented on top of each bar.

**Figure 3.**
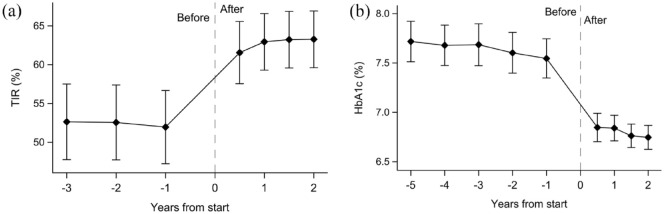
Time in range (a) and HbA1c (b) before and after start of AHCL therapy. Time in range levels were lower and HbA1c levels consistently elevated before treatment start with a rapid and sustained improvement during the follow-up period. Points and error bars represent means with 95% confidence intervals. Missing data after treatment intiation were handled using last observation carried forward.

During AHCL system therapy mean TIR increased by 14.5% from 57.0% to 71.5% (95% confidence interval [CI] = 12.2-16.9; *P* < .001). Mean TIT increased by 12.1% from 36.9% to 49.0% (95% CI = 9.3-15.0; *P* < .001) (Table 2). Eighty-seven (78%) and 71 (64%) individuals improved TIR by more than 5% and 10%, respectively (Figure 2). Temporal analysis showed that TIR was clearly suboptimal the year before starting treatment but then rapidly increased after pump initiation and was sustained at 2 years Figure 3a).

**Table 2.** Glycemic Outcomes Before and After Start of Advanced Hybrid Closed-Loop Therapy.

Variable	*n* subjects^a^	Baseline	End of follow-up	Mean difference (95% CI)	*P* value
Percentage of time in range, 70–180 mg/dL	136	57.0 (17.4)	71.5 (11.3)	14.5 (12.2 to 16.9)	< .001
Percentage of time in target, 70–144 mg/dL	104	36.9 (15.2)	49.0 (11.3)	12.1 (9.3 to 15.0)	< .001
Percent sensor time < 54 mg/dL	132	0.7 (2.0)	0.3 (0.7)	−0.5 (−0.9 to −0.1)	.023
Percent sensor time < 70 mg/dL	136	3.8 (4.6)	1.6 (1.8)	−2.2 (−3.0 to −1.4)	< .001
Percent sensor time > 180 mg/dL	135	39.4 (18.4)	26.9 (11.8)	−12.6 (−15.1 to −10.0)	< .001
Percent sensor time > 250 mg/dL	132	14.2 (14.3)	6.4 (5.9)	−7.8 (−10.2 to −5.3)	< .001
Time in range ≥70%, n (%)^b^	136	33 (24.5)	84 (61.8)	0.37 (0.28 to 0.47)^b^	< .001
HbA1c (%)	142	7.3 (1.1)	6.7 (0.7)	−0.6 (−0.8 to −0.5)	< .001
HbA1c ≤ 7%, n (%)^b^	142	55 (38.4)	96 (67.4)	0.29 (0.21 to 0.37)^b^	< .001
HbA1c ≥ 8%, n (%)^b^	142	34 (24.1)	7 (5.0)	−0.19 (−0.26 to −0.12)^b^	< .001
HbA1c ≥ 9%, n (%)^b^	142	14 (9.9)	2 (1.4)	−0.09 (−0.13 to −0.04)^b^	< .001
GMI (%)	136	7.4 (0.8)	7.0 (0.4)	−0.4 (−0.5 to −0.3)	< .001
Mean glucose (mg/dL)	136	170 (34.2)	154 (18.4)	−15.3 (−20.0 to −10.7)	< .001
Glucose variability SD (mg/dL)	136	61.1 (16.2)	51.4 (11.7)	−9.7 (−12.8 to −6.7)	< .001
Glucose variability CV (%)	110	35.4 (5.3)	32.9 (5.1)	−2.6 (−4.0 to −1.1)	< .001
Glycemia risk index	130	49.6 (23.7)	30.4 (13.4)	−19.2 (−23.2 to −15.3)	< .001
GRI, hypoglycemia component	132	2.6 (3.6)	1.3 (1.6)	−1.3 (−2.0 to −0.7)	< .001
GRI, hyperglycemia component	132	27.2 (16.7)	16.7 (8.6)	−10.5 (−13.2 to −7.7)	< .001

Descriptive data are presented using mean (standard deviation) for numeric variables and number (percentage) for categorical variables. Comparisons between baseline and end of follow-up were performed using repeated measures regression analysis, accounting for missing data.

Abbreviations: CI, confidence interval; DTSQ, Diabetes Treatment Satisfaction Questionnaire; GMI, glucose management indicator; GRI, glycemia risk index; HbA1c, glycated hemoglobin A1c; SD, standard deviation; CV, coefficient of variation.

a Number of subjects with data at baseline (last available measurement before treatment start) or follow-up (last available measurement after treatment start) included in the analyses.

b For binary variables (time in range ≥ 70% and HbA1c ≤ 7%, ≥ 8%, and ≥ 9%), the mean difference is the difference in proportions.

Glycated hemoglobin A1c decreased by 0.6% (6.8 mmol/mol) from 7.3% to 6.7% (56.6-49.8 mmol/mol; 95% CI = 0.5%-0.8%, 5.3-8.2 mmol/mol; *P* < .001). The number of patients reaching target HbA1c levels of ≤ 7.0% (≤ 53.0 mmol/mol) was increased by 29 percentage points, from 38.4% to 67.4% (*P* < .001; Table 2). Sensitivity analyses using data from complete cases showed consistent results (Supplementary Table S1). Fifty-seven percent of patients had improved HbA1c by more than 0.5% (5.5 mmol/mol; Figure 3a). Glycated hemoglobin A1c was persistently elevated 2 years prior to beginning use of AHCL systems with a subsequent rapid decrease post-initiation and, notably, lower HbA1c levels already after 3 months with effects sustained at 2 years (Figure 3b).

Mean TAR, > 250 mg/dL (> 13.9 mmol/L) decreased significantly from 14.2% to 6.4% (*P* < .001) and > 180 mg/dL (> 10 mmol/L) from 39.4% to 26.9% (*P* < .001). Mean TBR, < 70 mg/dL (< 3.9 mmol/L) decreased from 3.8% to 1.6% (*P* < .001) and < 54 mg/dL (< 3.0 mmol/L) from 0.7% to 0.3% (*P* = .023). Glycemic variability measured as SD and CV improved from 61 to 51 mg/dL (3.4-2.9 mmol/L; *P* < .001) and from 35.4% to 32.9% (*P* < .001), respectively. Mean glucose levels decreased from 170 to 154 mg/dL (9.4-8.6 mmol/L; *P* < .001). Similar results were obtained when analyses were restricted to complete cases (Supplementary Table S1).

No differences were found regarding change in TIR or other CGM metrics between the TCIQ and the M780 (Supplementary Table S2) groups. A larger reduction in HbA1c was found in patients using the TCIQ (baseline-adjusted mean difference −0.3% (−2.9 mmol/mol), 95% CI = −0.5% to −0.1% (−5.0 to −0.9 mmol/mol], *P* = .006). This difference remained when adjusting for age, sex, diabetes duration, and previous treatment with multiple daily insulin injections or insulin pump (*P* = .008).

### Adverse Events

Four cases of severe hypoglycemia occurred in four patients. Three of these patients were users of the M780 system. In patient “1,” hypoglycemia leading to unconsciousness occurred three days after activation of the SmartGuard function of the M780 system. Prior to this event the patient had reported completing a long walk and also administered a higher insulin dose relative to ingested meal after exercise. Patient “2” had a seizure secondary to hypoglycemia during use of the TCIQ system due to self-administrated insulin doses in addition to auto corrections given by the pump. By identical cause, that is, self-corrections by the patient, hypoglycemia leading to unconsciousness occurred in patient “3.” In patient “4,” hypoglycemia requiring assistance occurred during exercise.

Two patients reported long-lasting disabilities in forms of remaining scar tissue secondary to severe allergic reaction to adhesives used in Dexcom G6 CGM system (Dexcom, San Diego, CA, USA).

### Treatment Satisfaction and Insulin Pump Experience

Mean DTSQc score in the study population was 14.8 (SD = 3.5), indicating high treatment satisfaction overall, with significant improvements in all individual items (all *P* < .001, Supplementary Table S3). This was further affirmed by outcomes of the study-group-constructed questionnaire (Table 3). Patients in both treatment groups reported decreased fluctuations outside of TIR and subsequently decreased need for actively self-adjusting such fluctuations. Skin problems secondary to adhesive used in CGM devices and/or infusion sets were experienced by 42 (32%) patients with no significant difference between the two treatment groups (*P* = .90). Sensor problems were more common in patients treated with the M780 system than the TCIQ (mean [SD] = 4.7 [3.1] vs 3.1 [2.5], *P* = .006). This difference remained also when accounting for putative confounding factors (*P* = .002). There were no significant differences between the two treatment groups with regards to treatment satisfaction measured by DTSQs (*P* = .11) or DTSQc (*P* = .13) total scores (Supplementary Table S2).

**Table 3.** Outcomes Regarding Treatment Experience Using Questionnaires Constructed by the Authors.

Variable	Tandem Control IQ (*n* = 92)	MiniMed 780G (*n* = 50)	*P* value, unadjusted	*P* value, adjusted
Have you had skin problems due to adhesives used in the sensor or infusion set?	29 (31.5)	17 (34.0)	.90	.40
I am satisfied with my insulin pump	9.1 (1.0)	8.8 (1.8)	.79	.57
It is easy to change pump settings	8.2 (1.7)	8.4 (2.0)	.28	.15
I often experience sensor related problems	3.1 (2.5)	4.7 (3.1)	.006	.002
I estimate that I have (x) amount of sensor related problems per weak	1.3 (2.3)	1.0 (1.3)	.67	.25
I feel safe using my insulin pump	9.3 (0.9)	9.0 (1.6)	.54	.33
The sensor glucose value correlates well with capillary measurements	8.3 (1.3)	8.2 (1.4)	.77	.82
I have lower amounts of glucose values above 180 mg/dL since I started with my closed-loop pump	8.8 (1.7)	8.1 (2.6)	.26	.31
I have lower amounts of glucose values below 70 mg/dL since I started with my closed-loop pump	8.5 (1.8)	7.9 (2.5)	.31	.44
I do not need to correct glucose values above 180 mg/dL as often	8.1 (1.7)	8.0 (2.5)	.38	.54
I do not need to correct glucose values below 70 mg/dL as often	8.1 (2.1)	7.6 (2.5)	.42	.33
I have enough knowledge to maximize use of pump features	8.0 (1.8)	7.8 (1.9)	.68	.76
I have problems with insulin leaking from the needle inserted in the skin	2.2 (2.9)	1.6 (2.5)	.35	.23
I have problems with insulin delivery stopping inside the needle/tube or the pump signaling for occlusion	2.2 (2.7)	2.1 (2.6)	.76	.73
Because of insulin leakage I worry if administered insulin really goes into the body or not	2.2 (2.7)	1.8 (2.6)	.32	.20
I need to change insulin ampoule (reservoir) within (x) number of days	3.8 (1.8)	3.3 (1.2)	.032	.010
The insulin ampoule (reservoir) needs to be changed all too often	3.1 (3.1)	3.5 (3.6)	.78	.47

Scores ranged from 1 to 10, higher numbers indicating greater agreement with the statement given. Binary variables are reported as number (percentage). Numeric variables are reported as mean (standard deviation). Ordinal variables are treated as numeric in this regard. In unadjusted analyses, comparisons between groups were performed using Fisher’s exact test for binary variables and the Mann-Whitney *U* test for ordinal and numeric variables. Adjusted analyses were performed using Spearman’s partial correlation analyses, adjusting for age, sex, diabetes duration, and previous treatment with multiple daily injections or insulin pump.

### Post Hoc Analyses

Glycemia risk index improved by 19 points, from 49.6 to 30.4 (*P* < .001). The hypoglycemia component improved by 1.3 points, from 2.6 to 1.3 (*P* < .001), and hyperglycemia component by 10.5 points, from 27.2 to 16.7 (*P* < .001, Table 2). There were no differences between the TCIQ and the M780 systems with respect to glycemia risk index, hypoglycemia, or hyperglycemia component (Supplementary Table S2).

## Discussion

In this real-world evidence study, including both adverse events and practical experience of the TCIQ and M780 systems, mean TIR improved significantly and furthermore a reduction in time above and below targeted glucose range was observed. Glucose control improved within the first few months of treatment and was sustained throughout the follow-up period. Severe hypoglycemic episodes were rare, and all caused by self-administered correction boluses or during physical activity. High treatment satisfaction was reported by users in both treatment groups. Among reported side effects, skin problems were experienced by approximately one third of the study population.

TIR improved on average by 15 percentage units and was sustained over 1.7 years, which is substantial as improvement by 5 percentage units is considered clinically important.^21^ Notably, this exceeds TIR outcomes in trials conducted in more controlled clinical settings.^10,22^ In the pivotal study by Brown et al,^10^ use of the TCIQ system improved mean TIR from 61% at baseline to 71% at 6-month follow-up. Comparable improvements in TIR are found in several observational studies in real-world settings.^11,13,15^ One likely explanation for this marked improvement is poor glucose control prior to start of AHCL therapy with mean TIR in the TCIQ group being only 53.1% (SD = 17.6). Early onset of improvements in CGM metrics after treatment start is consistent with previous findings.^10,15,23,24^ Furthermore, glucose variability improved during AHCL system use, which has been demonstrated also in previous studies.^11,13,15^ Since glucose control substantially improves when switching to AHCL therapy in persons living with type 1 diabetes, the risk of long-term complications will likely decrease.^1-8^

TBR was reduced, generally decreasing the risk of severe hypoglycemia.^25^ The reported severe hypoglycemic events appearing in the current study occurred in conjunction with physical activity or extra manual insulin dosing by the patient. Since rapid-acting insulins used in insulin pumps last for several hours, a reduction in the basal insulin rate is, in many instances, not sufficient to avoid hypoglycemia in connection with physical activity. More rapid mealtime insulins will likely reduce this problem as already demonstrated in open-loop system users.^26^

On a larger scope, safety data of the TCIQ and M780 systems in real-world settings are sparse highlighting the need for further studies. In one prospective study by Messer et al,^13^ including 191 young adults and youths, no cases of severe hypoglycemia and two cases of ketoacidosis secondary to infusion set failure and viral gastroenteritis, respectively, occurred over 6 months use of the TCIQ™ system indicating high safety. This finding is supported by outcomes from several randomized controlled trials.^10,27,28^

Since around one third of patients had skin reactions from adhesives used in sensors and/or infusion sets and two reported long-lasting scars, it is essential with further research to develop more tolerable adhesives. Although more patients are receiving the TCIQ or the M780 system, it is important to note that even in developed nations most patients still use multiple daily insulin injections for insulin delivery.^29^ Furthermore, in a global perspective, most persons living with type 1 diabetes do not have access to CGM systems, which has been proven to reduce glucose levels in conjunction with MDI.^30^ Hence, an overall major challenge will be to make modern diabetes treatments more available for persons living with type 1 diabetes.

Current treatment satisfaction was increased dramatically after patients initiated AHCL therapy, shown by a score of 14.8 on a scale ranging from −18 (equivalent to higher satisfaction with the previous treatment) to 18 (equivalent to higher satisfaction with the current treatment). Ease of system use, high degree of perceived safety, reduction in fluctuations outside of targeted glucose range, and sensor accuracy are all potential contributing factors identified through the currently used questionnaire regarding insulin pump experience. Closed-loop systems have demonstrated a reduction in impact of diabetes on quality of life and increased treatment satisfaction in previous real-life studies with mean follow-up time of two to 12 months.^11,12^ Notably, our findings demonstrate increased treatment satisfaction after long-term use of AHCL systems.

Strengths of this study include the large sample of pre-AHCL glucose data (both HbA1c and CGM metrics), substantial follow-up time, and inclusion of PROs. Furthermore, the study was conducted independently of AHCL system manufacturers. Although we evaluated glucose metrics and PROs between the TCIQ and M780 systems, these findings must be interpreted with caution as it is generally difficult to compare effects of treatments in observational studies due to potential confounding. Furthermore, in the current study, considerably more patients had used the MiniMed 640G before starting use of the M780 than the TCIQ system. Detecting serious adverse events from a treatment being rare generally requires large patient populations. The questionnaire used regarding practical barriers during AHCL treatment was not validated but aimed at collecting essential information regarding practical experiences and treatment barriers and constructed before initiation of the study by nurses and physicians experienced in the clinical field of diabetes. Further future real-life evidence studies of AHCL systems are necessary particularly with respect to safety. Continued focus on safety is needed to avoid ketoacidosis as these can rapidly occur in case of a pump failure.

## Conclusions

The M780 and TCIQ systems should be considered for most persons with type I diabetes because they demonstrate early improvements in glucose control and treatment satisfaction while preserving a low risk profile. Thorough education in carbohydrate intake prior to and during physical activity should be provided. Furthermore, patients should be cautious with self-correcting high glucose levels as this can interfere with autocorrections given by the pump system leading to increased risk for severe hypoglycemia. Skin reaction due to adhesives used in sensors and/or infusion sets are common and patients should be informed of recommended precautions if such issues should occur. Novel more tolerable adhesives need to be developed. Making AHCL systems more available to persons with type 1 diabetes will likely substantially reduce long-term diabetes complications.

## Supplemental Material

sj-docx-1-dst-10.1177_19322968241242386 – Supplemental material for Effects, Safety, and Treatment Experience of Advanced Hybrid Closed-Loop Systems in Clinical Practice Among Adults Living With Type 1 Diabetes Supplemental material, sj-docx-1-dst-10.1177_19322968241242386 for Effects, Safety, and Treatment Experience of Advanced Hybrid Closed-Loop Systems in Clinical Practice Among Adults Living With Type 1 Diabetes by Ramanjit Singh, Henrik Imberg, Shilan Seyed Ahmadi, Sara Hallström, Johan Jendle, Bengt-Olov Tengmark, Anna Folino, Ekström Marie and Marcus Lind in Journal of Diabetes Science and Technology

sj-docx-2-dst-10.1177_19322968241242386 – Supplemental material for Effects, Safety, and Treatment Experience of Advanced Hybrid Closed-Loop Systems in Clinical Practice Among Adults Living With Type 1 Diabetes Supplemental material, sj-docx-2-dst-10.1177_19322968241242386 for Effects, Safety, and Treatment Experience of Advanced Hybrid Closed-Loop Systems in Clinical Practice Among Adults Living With Type 1 Diabetes by Ramanjit Singh, Henrik Imberg, Shilan Seyed Ahmadi, Sara Hallström, Johan Jendle, Bengt-Olov Tengmark, Anna Folino, Ekström Marie and Marcus Lind in Journal of Diabetes Science and Technology
