# Supplementary material for: Effects, Safety, and Treatment Experience of Advanced Hybrid Closed-Loop Systems in Clinical Practice Among Adults Living With Type 1 Diabetes
Source: J Diabetes Sci Technol. 2024 Apr 17;19(6):1598–607. doi: 10.1177/19322968241242386 (PMC11571990; doi:10.1177/19322968241242386)
Supplement: sj-docx-1-dst-10.1177_19322968241242386 – Supplemental material for Effects, Safety, and Treatment Experience of Advanced Hybrid Closed-Loop Systems in Clinical Practice Among Adults Living With Type 1 Diabetes [file sj-docx-1-dst-10.1177_19322968241242386.docx]

This supplement has been provided by the authors to offer additional information to their work.

Full title: Effects, Safety, and Treatment Experience of Advanced Hybrid Closed-Loop Systems in Clinical Practice Among Adults Living With Type 1 Diabetes

Authors: Ramanjit Singh^1,2^, Henrik Imberg^2,3,4^, Shilan Seyed Ahmadi^1,2^, Sara Hallström^1,2^, Johan Jendle^5^, Bengt-Olov Tengmark^6^, Anna Folino^7^, Marie Ekström^8^, and Marcus Lind^1,2,8^.

Author affiliations:

1. Department of Medicine, Sahlgrenska University Hospital, Gothenburg, Sweden
2. Department of Molecular and Clinical Medicine, Sahlgrenska Academy, University of Gothenburg, Gothenburg, Sweden.
3. Department of Mathematical Sciences, Chalmers University of Technology and University of Gothenburg, Gothenburg, Sweden
4. Statistiska Konsultgruppen, Gothenburg, Sweden
5. Faculty of Medicine and Health, School of Medical Science, Örebro University, Örebro, Sweden
6. Citydiabetes, Stockholm, Sweden
7. Department of Medicine and Emergency, Sahlgrenska University Hospital/Mölndal Hospital, Gothenburg, Sweden
8. Department of Medicine, NU Hospital Group, Uddevalla, Sweden

Corresponding author: Marcus Lind, Professor of Diabetology, Department of 
Molecular and Clinical Medicine, Wallenberg Laboratory, Bruna stråket 16, 413 45 Gothenburg, Sweden. e-mail: marcus.lind@gu.se, Tel: +46 (0)700 82 42 39.

Table S1. Glycemic outcomes before and after start of advanced hybrid closed-loop therapy in individuals with data at both baseline and follow-up (complete case analysis).

| Variable | n subjects | Baseline | End of  follow-up | Mean difference  (95% CI) | p-value |
| --- | --- | --- | --- | --- | --- |
| Percentage of time in range, 70–180 mg/dL | 111 | 56.4 [17.3] | 71.0 [11.2] | 14.6 (12.3 to 16.9) | <.001 |
| Percentage of time in target, 70–144 mg/dL | 59 | 37.3 [15.1] | 48.9 [11.2] | 11.6 (8.7 to 14.5) | <.001 |
| Percent sensor time <54 mg/dL | 81 | 0.7 [1.9] | 0.3 [0.9] | -0.4 (-0.8 to -0.0) | .049 |
| Percent sensor time <70 mg/dL | 107 | 3.8 [4.6] | 1.6 [1.9] | -2.2 (-3.0 to -1.4) | <.001 |
| Percent sensor time >180 mg/dL | 101 | 40.6 [18.5] | 28.0 [11.9] | -12.6 (-15.2 to -10.0) | <.001 |
| Percent sensor time >250 mg/dL | 75 | 14.2 [14.3] | 6.4 [5.8] | -7.8 (-10.4 to -5.2) | <.001 |
| Time in range ≥70%, n (%)^a^ | 111 | 26 (23.4) | 65 (58.6) | 0.35 (0.25 to 0.45)^a^ | <.001 |
| HbA1c (%) | 132 | 7.4 [1.1] | 6.7 [0.7] | -0.7 (-0.8 to -0.5) | <.001 |
| HbA1c ≤7%, n (%)^a^ | 132 | 47 (35.6) | 87 (65.9) | 0.30 (0.22 to 0.39)^a^ | <.001 |
| HbA1c ≥8%, n (%)^a^ | 132 | 34 (25.8) | 7 (5.3) | -0.20 (-0.28 to -0.13)^a^ | <.001 |
| HbA1c ≥9%, n (%)^a^ | 132 | 14 (10.6) | 2 (1.5) | -0.09 (-0.14 to -0.04)^a^ | <.001 |
| GMI (%) | 116 | 7.4 [0.8] | 7.0 [0.4] | -0.4 (-0.5 to -0.3) | <.001 |
| Mean glucose (mg/dL) | 116 | 170 [34.1] | 155 [18.2] | -15.4 (-20.1 to -10.6) | <.001 |
| Glucose variability SD (mg/dL) | 102 | 61.3 [16.3] | 51.9 [11.8] | -9.5 (-12.5 to -6.4) | <.001 |
| Glucose variability CV (%) | 58 | 35.4 [5.3] | 32.4 [4.7] | -3.0 (-4.5 to -1.4) | <.001 |
| Glycemia risk index | 75 | 49.8 [23.2] | 30.5 [12.7] | -19.3 (-23.3 to -15.3) | <.001 |
| GRI, hypoglycemia component | 81 | 2.6 [3.5] | 1.3 [1.8] | -1.2 (-1.9 to -0.6) | <.001 |
| GRI, hyperglycemia component | 75 | 27.0 [16.6] | 16.6 [8.5] | -10.4 (-13.2 to -7.6) | <.001 |
| Descriptive data are presented as mean [standard deviation] for numeric variables and number (percentage) for categorical variables.  Comparisons between baseline (last available measurement before treatment start) and end of follow-up (last available measurement after treatment start) were performed using paired T-test.  ^a^ For binary variables (time in range ≥70% and HbA1c ≤7%, ≥8% and ≥9%) the mean difference is the difference in proportions. Abbreviations: CI, confidence interval; GMI, glucose management indicator; GRI, glycemia risk index; HbA1c, glycated hemoglobin A1c; SD, standard deviation; CV, coefficient of variation. | | | | | |

Table S2. Change in glycemic outcomes and comparison of patient-reported outcomes in persons with type I diabetes initiating the MiniMed 780G and Tandem Control IQ system.

|  |  |  | Adjusted mean difference (95% CI) | |
| --- | --- | --- | --- | --- |
| Variable | Tandem  Control IQ | MiniMed 780G | Model 1 | Model 2 |
| Change in percent time in range 70–180 mg/dL | 16.2 [12.7] | 11.5 [11.2] | -0.5 (-3.8 to 2.8) p=.76 | 0.3 (-3.1 to 3.7) p=.85 |
| Change in percent time in target 70–144 mg/dL | 12.4 [12.1] | 10.5 [10.0] | -1.4 (-5.9 to 3.2) p=.54 | 0.4 (-4.5 to 5.3) p=.88 |
| Change in percent sensor time <54 mg/dL | -0.3 [1.5] | -0.6 [2.2] | 0.0 (-0.3 to 0.4) p=.84 | 0.2 (-0.2 to 0.6) p=.40 |
| Change in percent sensor time <70mg/dL | -2.5 [4.1] | -1.6 [4.3] | -0.6 (-1.3 to 0.0) p=.062 | -0.6 (-1.3 to 0.1) p=.091 |
| Change in percent sensor time >180 mg/dL | -13.5 [13.6] | -10.7 [11.6] | 1.6 (-2.1 to 5.2) p=.40 | 0.9 (-2.8 to 4.7) p=.63 |
| Change in percent sensor time >250 mg/dL | -9.8 [12.0] | -4.5 [8.6] | -0.3 (-2.3 to 1.8) p=.81 | -0.5 (-2.7 to 1.6) p=.63 |
| Change in HbA1c (%) | -0.7 [0.9] | -0.4 [0.6] | -0.3 (-0.5 to -0.1) p=.006 | -0.3 (-0.5 to -0.1) p=.008 |
| Change in GMI (%) | 0.4 [0.7] | -0.3 [0.5] | 0.1 (-0.1 to 0.2) p=.26 | 0.1 (-0.1 to 0.2) p=.43 |
| Change in mean glucose (mg/dL) | -17.6 [27.6] | -10.7 [20.7] | 3.1 (-2.3 to 8.5) p=.26 | 2.2 (-3.4 to 7.9) p=.43 |
| Change in glucose variability SD (mg/dL) | 11.7 [16.6] | -4.4 [12.5] | 1.0 (-3.9 to 5.9) p=.70 | 1.4 (-3.7 to 6.6) p=.58 |
| Change in glucose variability CV (%) | -3.1 [6.2] | -2.8 [5.5] | 0.4 (-2.1 to 2.9) p=.75 | 1.0 (-1.6 to 3.6) p=.43 |
| Change in glycemia risk index | -22.4 [17.1] | -14.1 [16.8] | -0.7 (-5.3 to 4.0) p=.78 | -1.3 (-6.0 to 3.5) p=.60 |
| Change in GRI, hypoglycemia component | -1.2 [2.6] | -1.2 [3.2] | -0.3 (-1.0 to 0.4) p=.39 | -0.0 (-0.8 to 0.7) p=.92 |
| Change in GRI, hyperglycemia component | -12.2 [13.2] | -7.3 [9.9] | -0.0 (-3.0 to 3.0) p=.99 | -1.3 (-4.3 to 1.8) p=.42 |
| DTSQ, status version | 32.3 [3.0] | 31.0 [5.0] | 1.3 (-0.3 to 2.9)  p=.11 | 1.3 (-0.3 to 3.0) p=.11 |
| DTSQ, change version | 15.2 [3.0] | 14.1 [4.3] | 1.1 (-0.3 to 2.4)  p=.13 | 1.2 (-0.3 to 2.7) p=.11 |
| Descriptive data are presented as mean [standard deviation].  Model 1: Analyses of change variables were performed using analysis of covariance, adjusting for baseline values. DTSQ was analyzed using Welch’s T-test.  Model 2: Additionally adjusted for age, sex, diabetes duration, and previous treatment with multiple daily injections or insulin pump. Abbreviations: CI, confidence interval; DTSQ, diabetes treatment satisfaction questionnaire; GMI, glucose management indicator; GRI, glycemia risk index; HbA1c, glycated hemoglobin A1c; SD, standard deviation; CV, coefficient of variation. | | | | |

Table S3. Diabetes treatment satisfaction questionnaire, change version (DTSQc), with advanced hybrid closed-loop therapy compared to previous treatment (n=141).

| Item | Mean [SD] | Range | 95% CI for mean | p-value |  |
| --- | --- | --- | --- | --- | --- |
| 1. How satisfied are you with your current treatment? | 2.7 [0.6] | 0 to 3 | 2.6 to 2.8 | <.001 |  |
| 2. How often have you felt that your blood sugars have been unacceptably high recently? | -1.4 [1.6] | -3 to 2 | -1.7 to -1.2 | <.001 |  |
| 3. How often have you felt that your blood sugars have been unacceptably low recently? | -1.7 [1.3] | -3 to 3 | -1.9 to -1.5 | <.001 |  |
| 4. How convenient have you been finding your treatment to be recently? | 2.4 [1.1] | -3 to 3 | 2.2 to 2.5 | <.001 |  |
| 5. How flexible have you been finding your treatment to be recently? | 2.2 [1.1] | -3 to 3 | 2.0 to 2.4 | <.001 |  |
| 6. How satisfied are you with your understanding of your diabetes? | 2.0 [1.0] | 0 to 3 | 1.8 to 2.1 | <.001 |  |
| 7. Would you recommend this form of treatment to someone else with your kind of diabetes? | 2.8 [0.7] | -2 to 3 | 2.6 to 2.9 | <.001 |  |
| 8. How satisfied would you be to continue with your present form of treatment? | 2.8 [0.5] | 0 to 3 | 2.8 to 2.9 | <.001 |  |
| DTSQc total scale | 14.8 [3.5] | -6 to 18 | 14.2 to 15.4 | <.001 |  |
| Statistical analyses were performed using the one-sample T-test.  Each item in DTSQc ranges from -3 to 3. Item 1 and 4–8: larger indicates higher treatment satisfaction with the current treatment. Item 2–3: smaller indicates higher treatment satisfaction with the current treatment. Total scale: sum of item 1 and item 4–8. | | | | | |
